# Architecture of the Serotonergic Projectome in the Mouse Brain

**DOI:** 10.64898/2026.07.15.738594

**Published:** 2026-09-03

**Authors:** Jun Ho Song, Drew Friedmann, Yunming Wu, Ashley Moses, Jalal Kenji Baruni, Yuan Yuan, Isabella Rana, Kelly Zhao, Tom Hindmarsh Sten, Qian Wang, Shuyun Alina Xiao, Xiaoke Chen, Scott Warren Linderman, Liqun Luo

## Abstract

The serotonin system innervates nearly the entire brain to support diverse functions, and its dysfunction is implicated in multiple psychiatric disorders. Although recent studies suggested that this anatomically diffuse system supports differentiated rather than uniform modulation, its overall organization is unclear. Here, using systematic whole-brain axon tracing and integrated analyses, we show that the serotonin projectome is organized by functional relatedness rather than physical proximity of its targets. Dorsal and median raphe serotonin neurons partition into five projectomic groups, each preferentially innervating the hippocampus, basal ganglia, cortex, medial interbrain, or brainstem/lateral thalamus, with within-group structure ranging from discrete subgroups to continuous variation. Spatial transcriptomic analyses reveal that each projectomic group corresponds to a distinct combination of transcriptomic clusters, with transcriptome and somatic position jointly predicting projectomic identity. Projection-specific serotonin depletion produces dissociable behavioral phenotypes. Together, projectomic identity emerges as a central axis linking axon collateralization, molecular diversity, and behavioral function.

## Full Text Availability

The license terms selected by the author(s) for this preprint version do not permit archiving in PMC. The full text is available from the preprint server.

