# Supplementary material for: Projectomic Organization of the Serotonin System of the Mouse Brain
Source: bioRxiv. 2026 Jul 17:2026.07.15.738594. Preprint. [Version 1] doi: 10.64898/2026.07.15.738594 (PMC13404724; doi:10.64898/2026.07.15.738594)
Supplement: 1 [file NIHPP2026.07.15.738594v1-supplement-1.pdf]

bioRxiv preprint doi: https://doi.org/10.64898/2026.07.15.738594; this version posted July 17, 2026. The copyright holder for this preprint (which was not certified by peer review) is the author/funder, who has granted bioRxiv a license to display the preprint in perpetuity. It is made available under a CC-BY 4.0 International license.

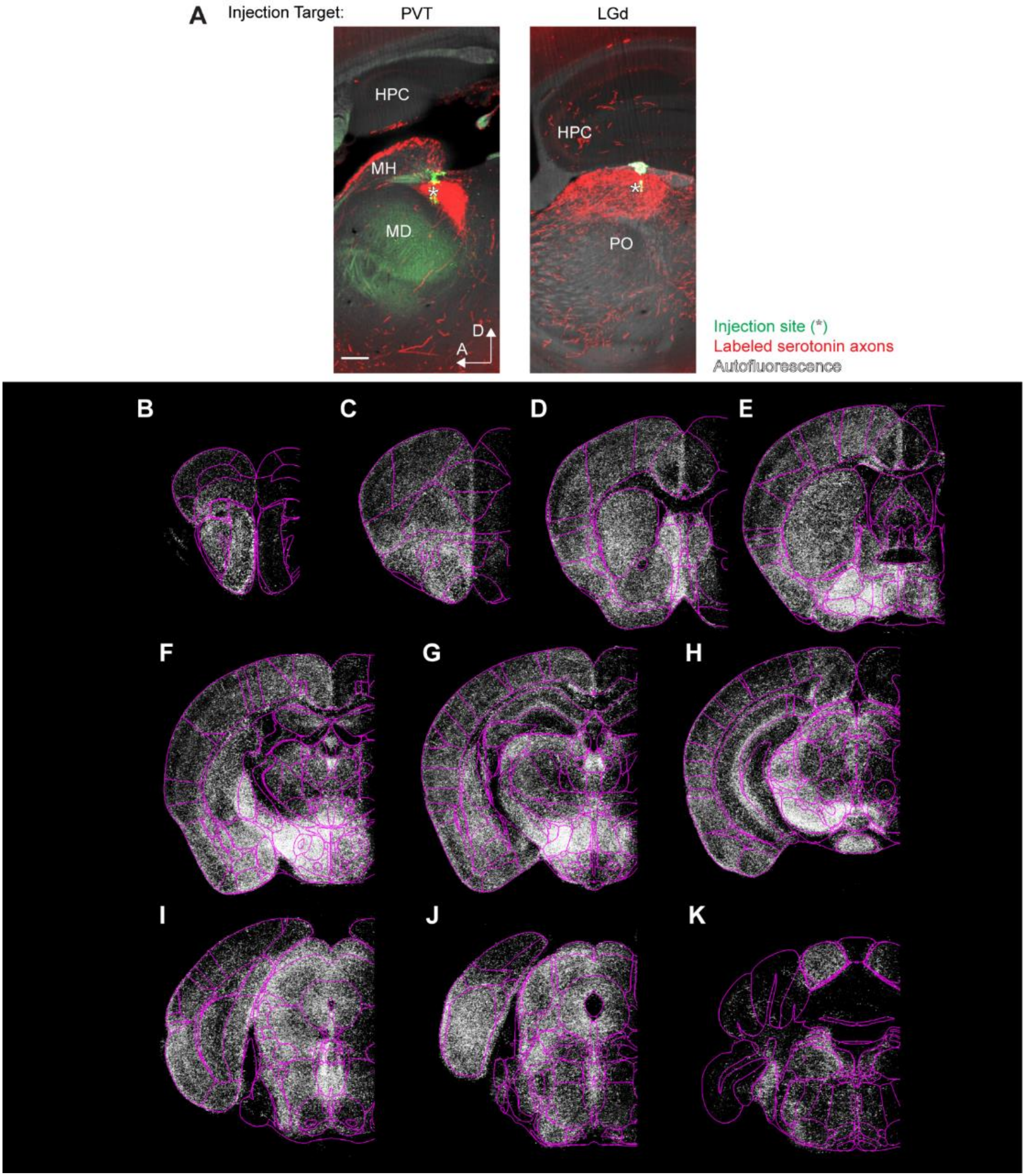

Figure S1. Validation of injection specificity and whole-brain axon coverage of the dataset, related to Figure 1

(A) Potential virus leakage and spillover into brain regions adjacent to the intended injection sites were examined. For example, the mediodorsal nucleus of the thalamus (MD), a region neighboring the paraventricular nucleus of the thalamus (PVT), shows little axon labeling in samples where the virus was injected into the PVT (left panel). Similarly, the posterior complex of the thalamus (PO) exhibits no dense labeling in samples injected into

bioRxiv preprint doi: https://doi.org/10.64898/2026.07.15.738594; this version posted July 17, 2026. The copyright holder for this preprint (which was not certified by peer review) is the author/funder, who has granted bioRxiv a license to display the preprint in perpetuity. It is made available under a CC-BY 4.0 International license.

the dorsal part of the lateral geniculate complex (LGd; right panel). These data confirm that the viral injections remained mostly confined to their designated targets with minimal unintended transduction in neighboring brain regions. Red retrobeads, pseudocolored green, were mixed with the virus at a 1:25 ratio to mark the injection sites. Scale bar: 200 μm; Section thickness: 30 μm; maximal intensity projection. HPC, hippocampus; MH, medial habenula.

(B–K) Overlay of axons segmented from all 110 sample brains in the dataset (white). Example coronal slices were collected along the anterior-posterior (AP) axis at 300–500 μm intervals. Magenta outlines denote the boundaries of brain regions used in analyses. Note that the contralateral hemisphere (right) was not fully imaged due to the working depth limitations of the objective used.

bioRxiv preprint doi: https://doi.org/10.64898/2026.07.15.738594; this version posted July 17, 2026. The copyright holder for this preprint (which was not certified by peer review) is the author/funder, who has granted bioRxiv a license to display the preprint in perpetuity. It is made available under a CC-BY 4.0 International license.

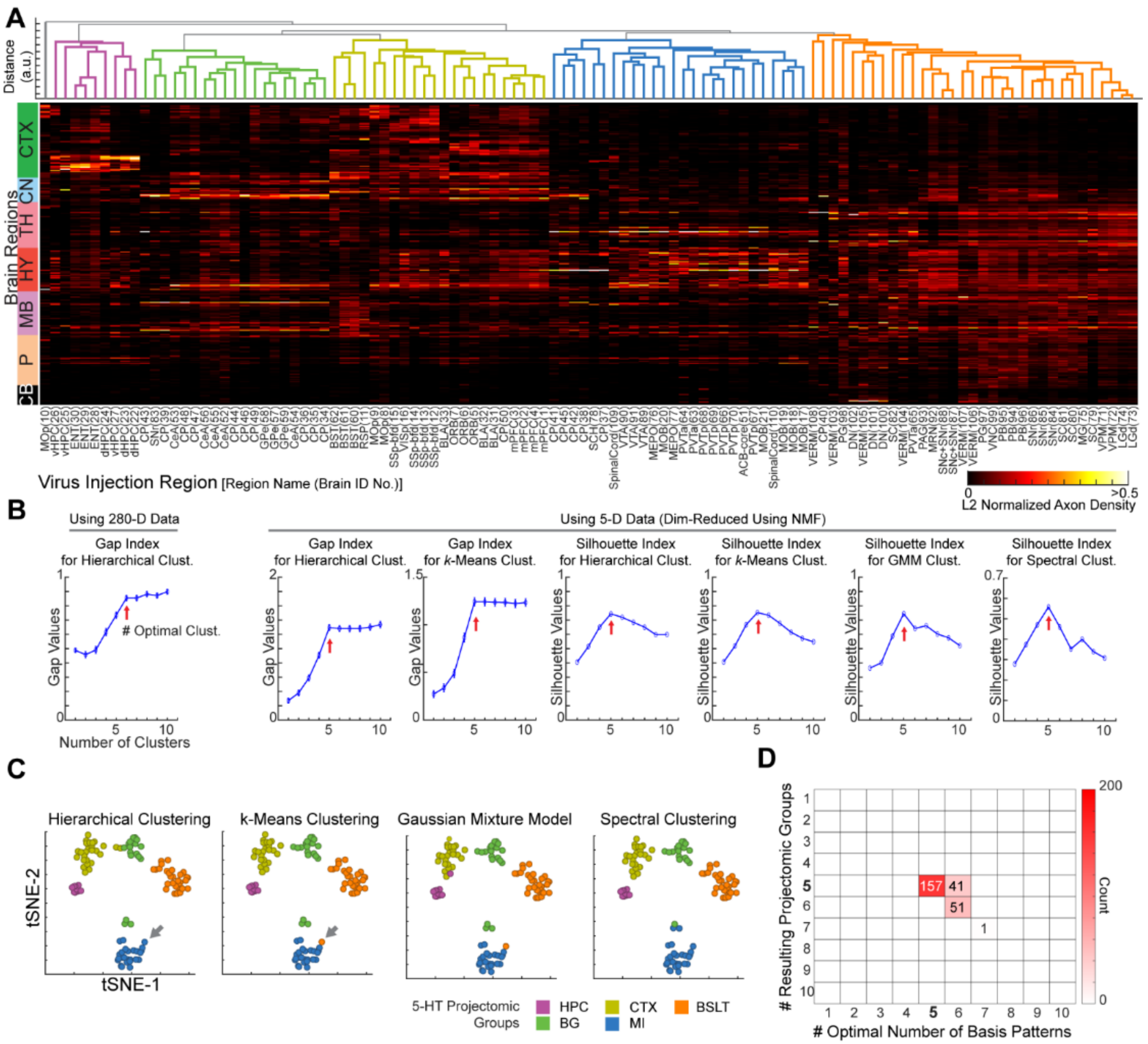

Figure S2. Evaluation of the stability of the NMF-based analysis, related to Figure 2

(A) Hierarchical clustering using the 280-D axon density data shown in **Figure 1D**. Hierarchical clustering was performed on samples in the original dimensional space (110 brains) to evaluate whether the clustering results shown in **Figure 2E**, based on NMF-dimension-reduced samples, accurately reflect clustering using 280-D normalized axon density data. Gap statistics identified 6 as the optimal number of clusters (panel B, leftmost plot). Hierarchical clustering into 6 clusters yielded 5 primary clusters and 1 "noise" cluster containing only one sample (the leftmost brain). Most samples (91.8%; 101 brains) were assigned to the same groups as in **Figure 2E**.

(B) Determining the optimal number of clusters for classifying brain samples into projectomic groups. Gap and silhouette indices were used to analyze the samples and identify the optimal number of clusters (N) for various clustering methods. The analysis was conducted on the dataset in its original dimension (280-D) for the leftmost panel and on the NMF-dimension-reduced dataset for the remaining panels. For silhouette index-based analysis[120], the number of clusters that produced the highest silhouette value was chosen. For gap index-based analysis[116], we used the criterion that gap(N) must be $\geq$ [gap(N+1) – standard error(N+1)] for N to be selected.

bioRxiv preprint doi: https://doi.org/10.64898/2026.07.15.738594; this version posted July 17, 2026. The copyright holder for this preprint (which was not certified by peer review) is the author/funder, who has granted bioRxiv a license to display the preprint in perpetuity. It is made available under a CC-BY 4.0 International license.

Once the incremental gain from increasing N falls within the standard error—reflecting the random variability in the gap statistic—the improvement is considered negligible, and N is taken as optimal. We note that the optimal number of clusters consistently remained at 5 for the dimensionality-reduced dataset across different combinations of optimal cluster number criteria and clustering techniques. The error bars in the gap index plots represent the standard error of the means. GMM, Gaussian mixture model.

(C) To evaluate the variability in clustering results using different clustering methods described in panel B, brain samples were visualized in 2-D plots. The leftmost plot (data from **Figure 2E**) shows results based on hierarchical clustering, and the outcomes from three other clustering methods are also represented using the same tSNE axes. As is evident, most brains consistently fall into the same group across all clustering methods. For example, the hierarchical- and *k*-means clustering methods differ by a single brain (arrow), which is assigned to the MI group by hierarchical clustering but to the BSLT group by *k*-means clustering.

(D) Testing the sensitivity of clustering results to sampling. To evaluate the stability of the results shown in Figure 2 to specific samples in our dataset, a random 22 samples (20% of the 110 samples) were removed, and the analysis was conducted on the remaining 88 samples for each trial; this was repeated 250 times. The optimal numbers of basis patterns and clusters are summarized in the graph. Most trials (79.2%, 198 trials) yielded five projectomic groups, with 62.8% (157 trials) yielded five basis patterns and five projectomic groups.

bioRxiv preprint doi: https://doi.org/10.64898/2026.07.15.738594; this version posted July 17, 2026. The copyright holder for this preprint (which was not certified by peer review) is the author/funder, who has granted bioRxiv a license to display the preprint in perpetuity. It is made available under a CC-BY 4.0 International license.

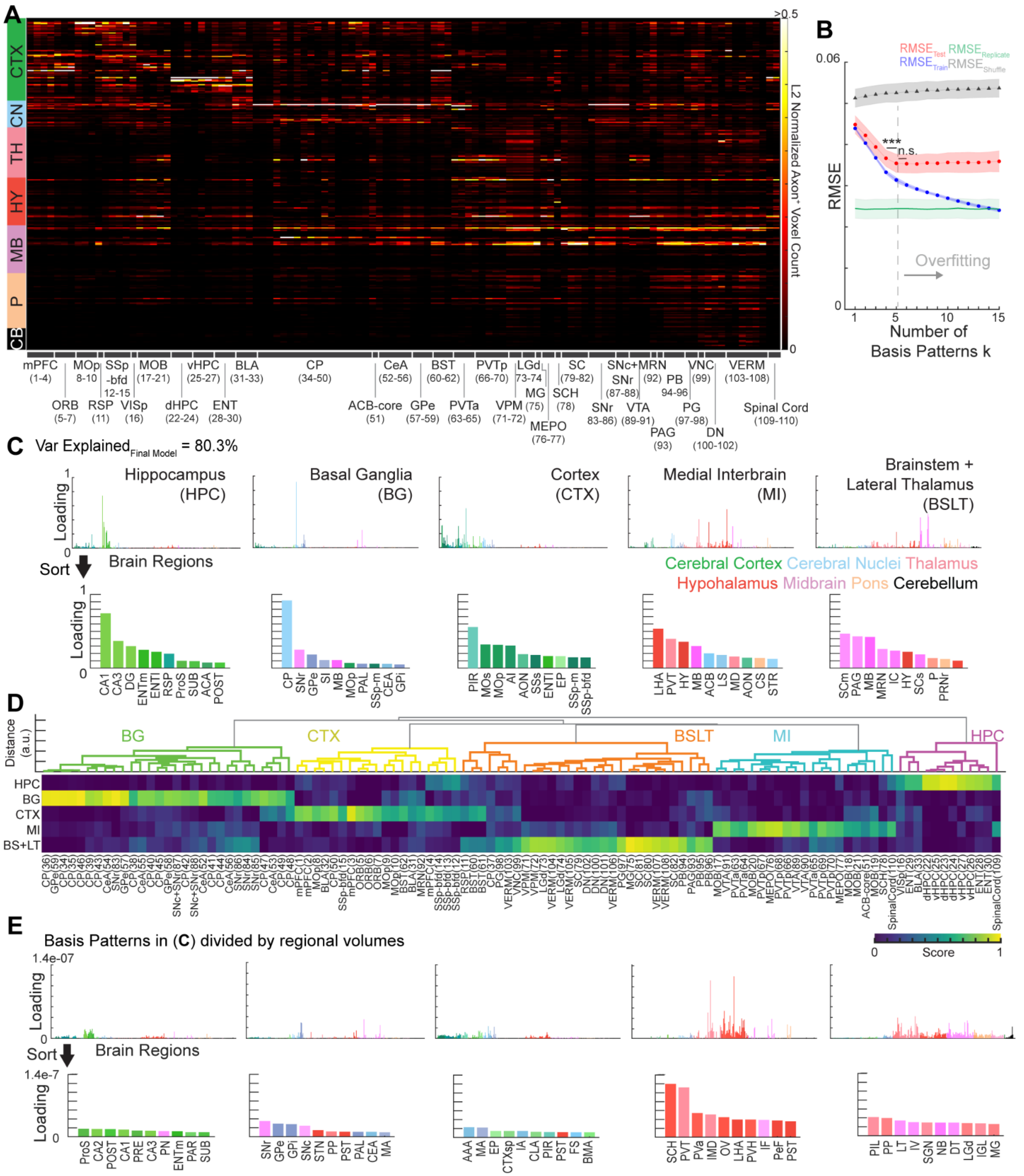

Figure S3. Regional axonal volumes–based NMF analysis, related to Figure 2

(A) Data from **Figure 1D**, originally plotted using normalized regional axon density, is replotted here using normalized regional axon volumes, representing the total quantity of axons in individual brain regions.

(B–D) NMF analysis based on normalized regional axon volume data. Cross-validation results again indicated that the dataset dimension could be optimally reduced to k = 5 (**B**), and the projection basis patterns captured

bioRxiv preprint doi: https://doi.org/10.64898/2026.07.15.738594; this version posted July 17, 2026. The copyright holder for this preprint (which was not certified by peer review) is the author/funder, who has granted bioRxiv a license to display the preprint in perpetuity. It is made available under a CC-BY 4.0 International license.

anatomical characteristics (**C**). Hierarchical clustering results (**D**) were largely consistent with those in **Figure 2E**, though some differences were observed. For example, SNr-injected brains were now classified within the BG group, whereas most were assigned to the BSLT group in the density-based analysis. Overall, 84.6% (93) brains were classified in the same groups as in **Figure 2E**.

(E) The basis patterns shown in panel C were divided by regional volumes, yielding results similar to those obtained from the density-based analysis shown in **Figure 2D**.

bioRxiv preprint doi: https://doi.org/10.64898/2026.07.15.738594; this version posted July 17, 2026. The copyright holder for this preprint (which was not certified by peer review) is the author/funder, who has granted bioRxiv a license to display the preprint in perpetuity. It is made available under a CC-BY 4.0 International license.

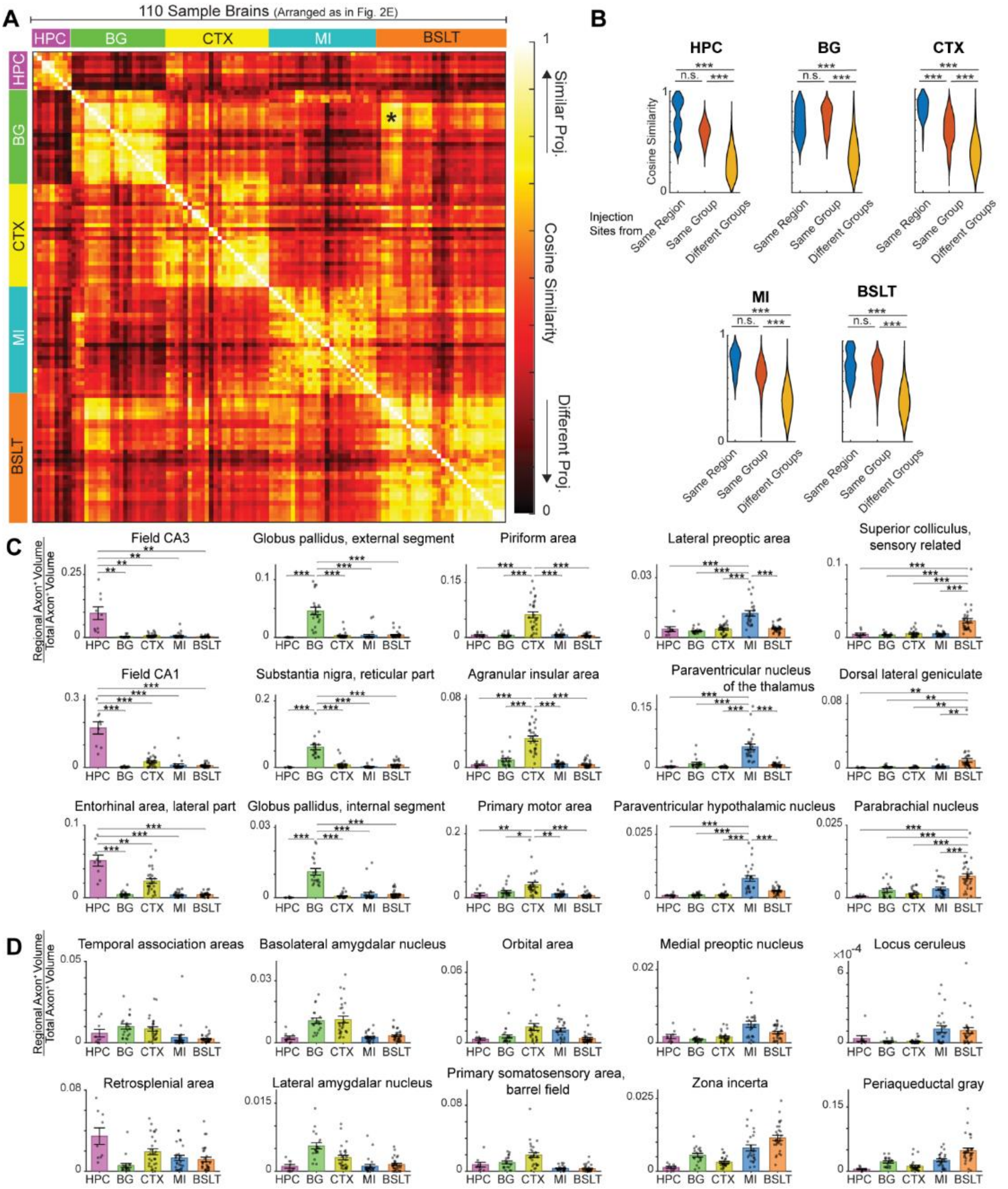

Figure S4. Projection similarity analysis supports NMF-based grouping and identifies marker regions, related to Figure 2

(A) Pairwise similarity was measured using cosine similarity on the 280-D dataset shown in **Figure 1D**. The brain sample order follows the clustering order presented in **Figure 2E**. The brains in the BSLT group forming

bioRxiv preprint doi: https://doi.org/10.64898/2026.07.15.738594; this version posted July 17, 2026. The copyright holder for this preprint (which was not certified by peer review) is the author/funder, who has granted bioRxiv a license to display the preprint in perpetuity. It is made available under a CC-BY 4.0 International license.

the off-diagonal "island" (marked with *) were injected into SNr and SNc+SNr and showed high similarity to some CeA- and GPe-injected brains in the BG group.

(B) Pairwise similarity measured between brains injected in the same regions (blue), different regions in the same projectomic groups (orange), and different regions in other projectomic groups (yellow) were statistically compared. Kruskal-Wallis test with Dunn's post-hoc correction. ***, $p < 0.001$; n.s., not statistically significant.

(C) Normalized axonal projection density (fraction of total axonal output per brain) across five projectomic groups (HPC, BG, CTX, MI, BSLT; n = 9, 19, 27, 25, 30 brain samples, respectively). Bars, mean ± SEM; points, individual mice. Marker regions were defined by three criteria: (1) significant group effect by one-way ANOVA ($p < 0.05$); (2) the highest-mean group exceeding twice the mean of every other group; and (3) Welch's two-sided t-tests of the highest-mean group versus each of the other four groups all significant after within-region BKY FDR correction ($p < 0.05$). *$p < 0.05$, **$p < 0.01$, ***$p < 0.001$.

(D) Examples of regions showing relatively high axonal projection density in more than one projectomic group. Normalized axonal projection density across five projectomic groups. These regions did not meet the marker-region criteria in (C); specifically, they had at least two groups whose mean exceeded half of the highest group mean.

bioRxiv preprint doi: https://doi.org/10.64898/2026.07.15.738594; this version posted July 17, 2026. The copyright holder for this preprint (which was not certified by peer review) is the author/funder, who has granted bioRxiv a license to display the preprint in perpetuity. It is made available under a CC-BY 4.0 International license.

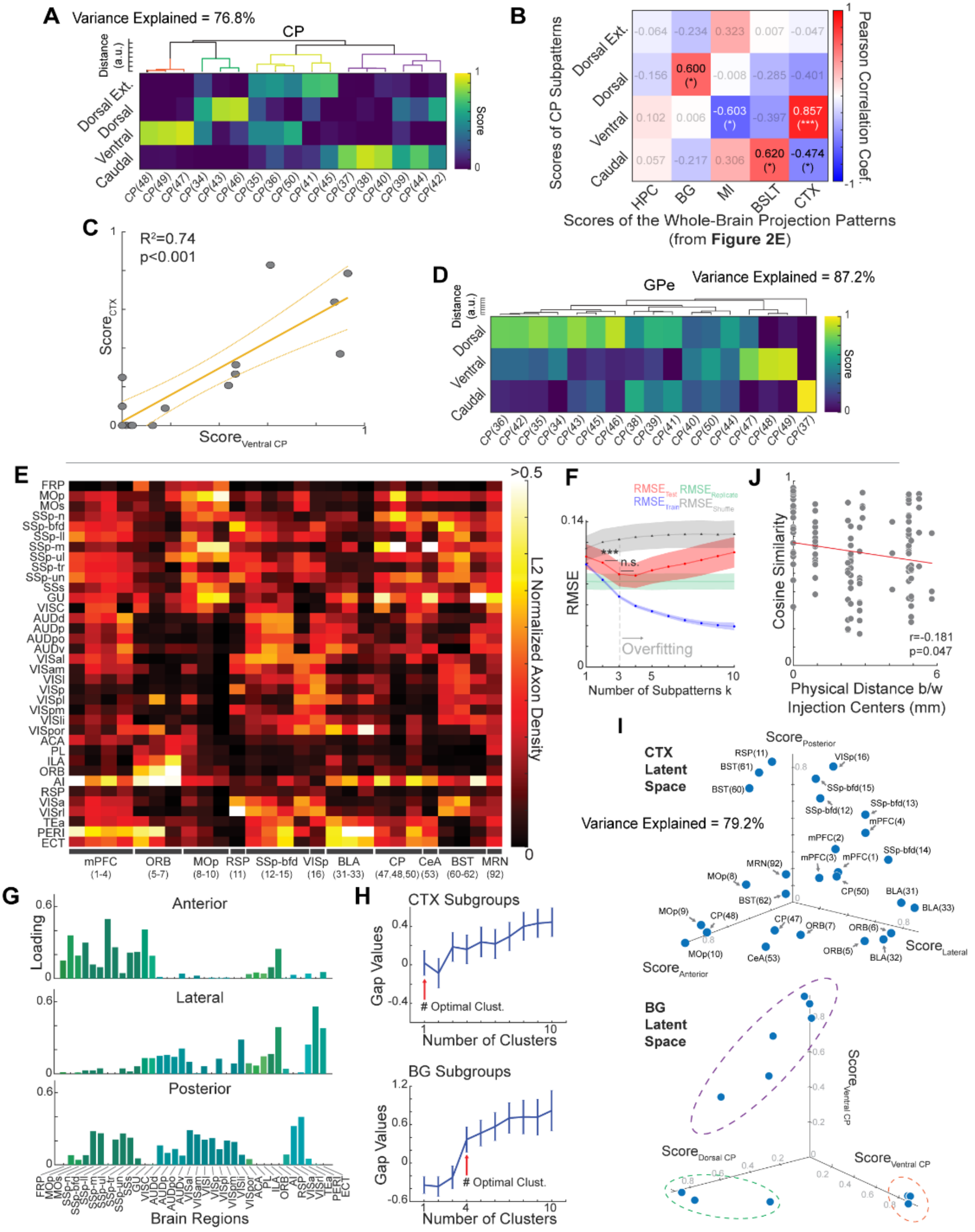

Figure S5. Analysis of basal ganglia and cortical subpatterns, related to Figure 3

bioRxiv preprint doi: https://doi.org/10.64898/2026.07.15.738594; this version posted July 17, 2026. The copyright holder for this preprint (which was not certified by peer review) is the author/funder, who has granted bioRxiv a license to display the preprint in perpetuity. It is made available under a CC-BY 4.0 International license.

(A) Axon distributions of CP-injected brains were dimensionality-reduced and hierarchically clustered using the 4D scores. Subgroups were visually distinguished by assigning unique colors to each in the dendrogram. The corresponding subgroups were enclosed within color-matching dotted circles in the bottom of Panel I.
(B) Pairwise Pearson correlation between the scores of CP subpatterns from Panel A and scores of the whole-brain projection patterns of the 17 CP-injected brains (from **Figure 2E**). By extending our analysis to examine whole-brain serotonergic innervation using the scores of the CP-injected brains shown in **Figure 2E**, we identified statistically significant correlations between several CP subpatterns and the main projectomic patterns. *, $p < 0.05$; ***, $p < 0.001$; gray values indicate no statistical significance.
(C) Linear regression between the ventral CP subpattern scores and the CTX pattern scores of the 17 CP-injected brains. The near-zero *y*-intercept and high R^2 value indicates that serotonergic axon labeling in the cortex of CP-injected brains was primarily driven by the labeling of serotonergic axons targeting the ventrolateral CP.
(D) Hierarchical clustering of GPe axon projection patterns from the brains with injections in CP. High-dimensional axon distributions were first decomposed into a low-dimensional space using the basis patterns in **Figure 3E**, and the resulting scores were hierarchically clustered.
(E) L2-normalized serotonergic axon density in the isocortical regions of CTX group samples (n = 27). Injection sites are listed on the *x*-axis, and cortical projection regions are listed on the *y*-axis.
(F) Speckled cross-validation of the axon density matrix shown in panel A indicates that the optimal number of CTX subpatterns is 3. For each iteration, 10% of randomly selected entries were used as test sets and 90% as training sets. 2500 iterations. One-way ANOVA followed by Bonferroni correction. Error bars, standard deviation. ***, $p < 0.001$; n.s., not statistically significant.
(G) Three extracted CTX basis subpatterns showing their loadings across cortical areas.
(H) Gap indices were applied to identify the optimal number of subgroups within the CTX (and BG groups as a comparison) using hierarchical clustering (top and bottom panels, respectively). See **Figure S2B** for details on the gap index criteria.
(I) 3D loadings of the dimensionality-reduced individual brains in the CTX group (top) and BG group (bottom, as a comparison) are shown in their respective latent space. CTX samples are widely distributed across the latent space and do not form discrete clusters, consistent with Gap analysis suggesting a single cluster (panel H). This contrasts with the clustering observed in the BG samples, with three subgroups outlined by dashed lines colored according to hierarchical clustering in panel A. The Dorsal-Extreme subgroup is omitted for display purpose. Note that, in the CTX-sample distribution, all three basolateral amygdala (BLA)-injected brains had high scores on the lateral subpattern, like orbitofrontal cortex (ORB)-injected brains, whereas two of the three CP-injected brains had high scores on the anterior subpattern like primary motor cortex (MOp)-injected brains.
(J) Pairwise cosine similarity and the Euclidean distance of injection target region centers exhibit a weak but significant negative linear correlation. Data are from the 16 cortex-injected samples in Panel E. Pearson correlation was used for this analysis.

bioRxiv preprint doi: https://doi.org/10.64898/2026.07.15.738594; this version posted July 17, 2026. The copyright holder for this preprint (which was not certified by peer review) is the author/funder, who has granted bioRxiv a license to display the preprint in perpetuity. It is made available under a CC-BY 4.0 International license.

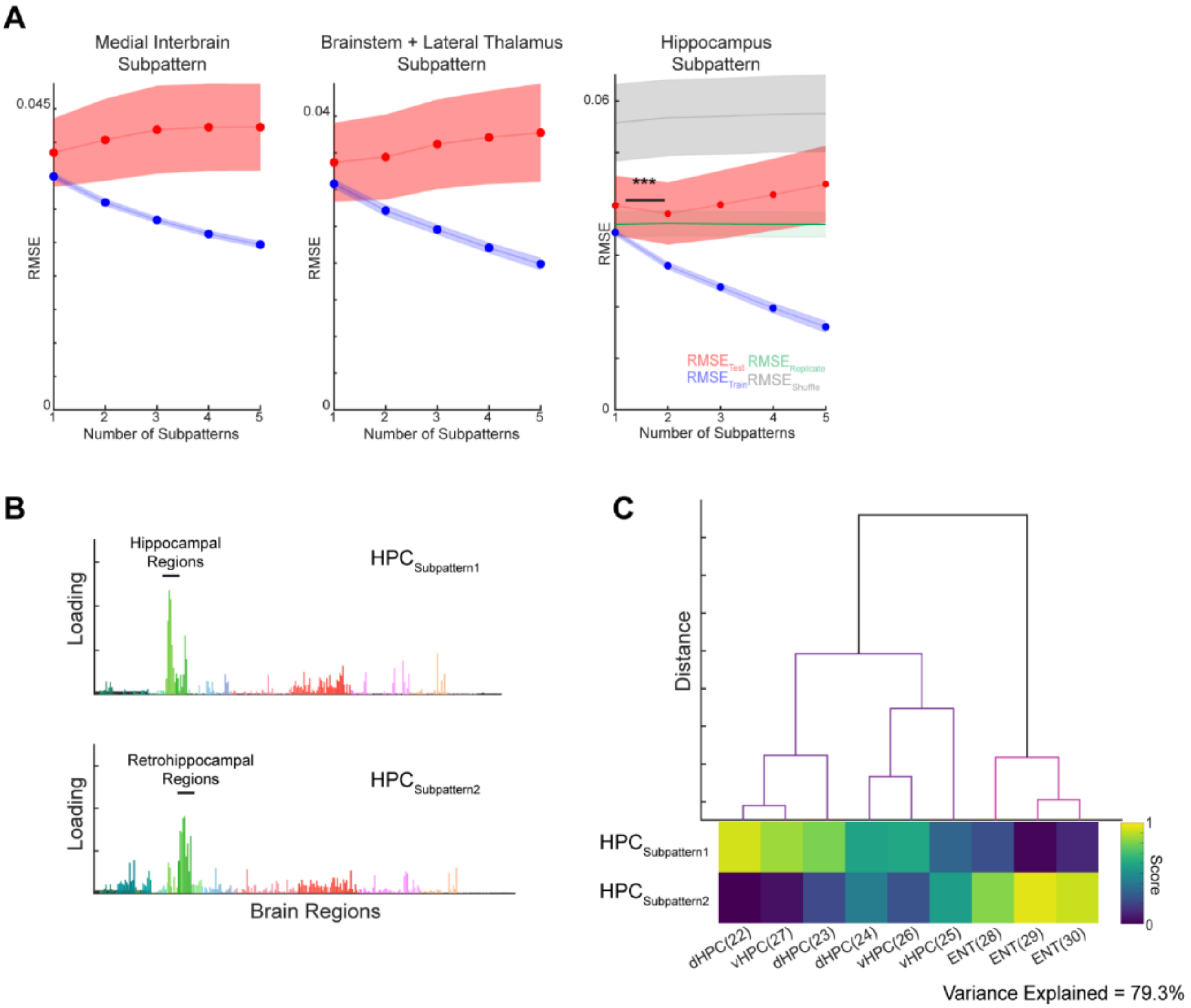

Figure S6. Subpattern analysis of MI, BSLT, and HPC patterns, related to Figure 3

(A) The same analytical pipeline from **Figure S5** was applied to the MI, BSLT, and HPC patterns. Whereas the NMF-based approach did not identify any distinct subpatterns within the MI and BSLT patterns, the HPC pattern can be divided into two subpatterns. 2500 iterations. One-way ANOVA followed by Bonferroni correction. Error bars, standard deviation. ***, $p < 0.001$.

(B) The two HPC subpatterns exhibited high loadings in distinct regions: one in the hippocampal regions (e.g., CA1, CA2, CA3) and the other in retrohippocampal regions (e.g., entorhinal cortex).

(C) Brains from the HPC projectomic group are hierarchically clustered based on these two identified subpatterns.

bioRxiv preprint doi: https://doi.org/10.64898/2026.07.15.738594; this version posted July 17, 2026. The copyright holder for this preprint (which was not certified by peer review) is the author/funder, who has granted bioRxiv a license to display the preprint in perpetuity. It is made available under a CC-BY 4.0 International license.

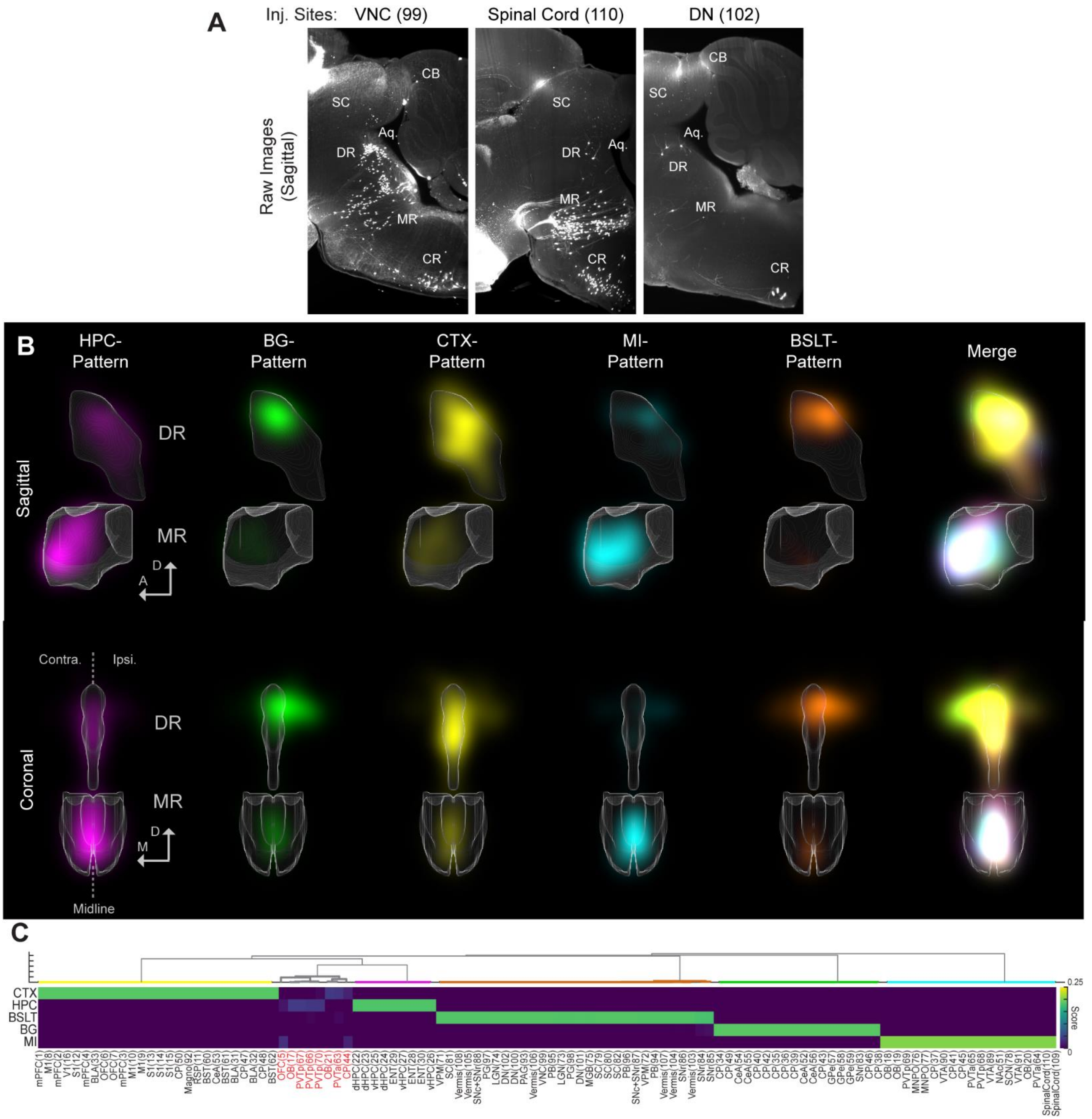

Figure S7. Distributions of cell body positions of serotonin neurons within DR and MR, related to Figure 4

(A) Example brains showing labeled serotonergic cell bodies in the caudal raphe (CR). Although we observed labeled serotonin neurons in CR in the samples injected in the hindbrain and spinal cord, they were not fully analyzed in this study, as we did not image the full distributions of these cells due to the limited imaging window size.

(B) Supervised NMF was used to decompose cell body distributions in the DR and MR into a set of basis patterns. The optimal number of patterns was determined to be five via a hyperparameter search using Bayesian optimization with 5-fold cross-validation, a result consistent with the number of projectomic patterns. The classification accuracy reported in **Figure 4C** was evaluated using 5-fold stratified cross-validation with these

bioRxiv preprint doi: https://doi.org/10.64898/2026.07.15.738594; this version posted July 17, 2026. The copyright holder for this preprint (which was not certified by peer review) is the author/funder, who has granted bioRxiv a license to display the preprint in perpetuity. It is made available under a CC-BY 4.0 International license.

optimized hyperparameters. For visualization, the basis patterns shown here and the sample scores in (**C**) are from a model trained on all 110 samples using the same optimized hyperparameters.

(C) Cell body distributions in individual sample brains represented using the basis distribution patterns. Some of the brains—brain ID numbers 5, 17, 67, 66, 70, 21, 63, and 44 (labeled in red)—were not adequately described by our model.

bioRxiv preprint doi: https://doi.org/10.64898/2026.07.15.738594; this version posted July 17, 2026. The copyright holder for this preprint (which was not certified by peer review) is the author/funder, who has granted bioRxiv a license to display the preprint in perpetuity. It is made available under a CC-BY 4.0 International license.

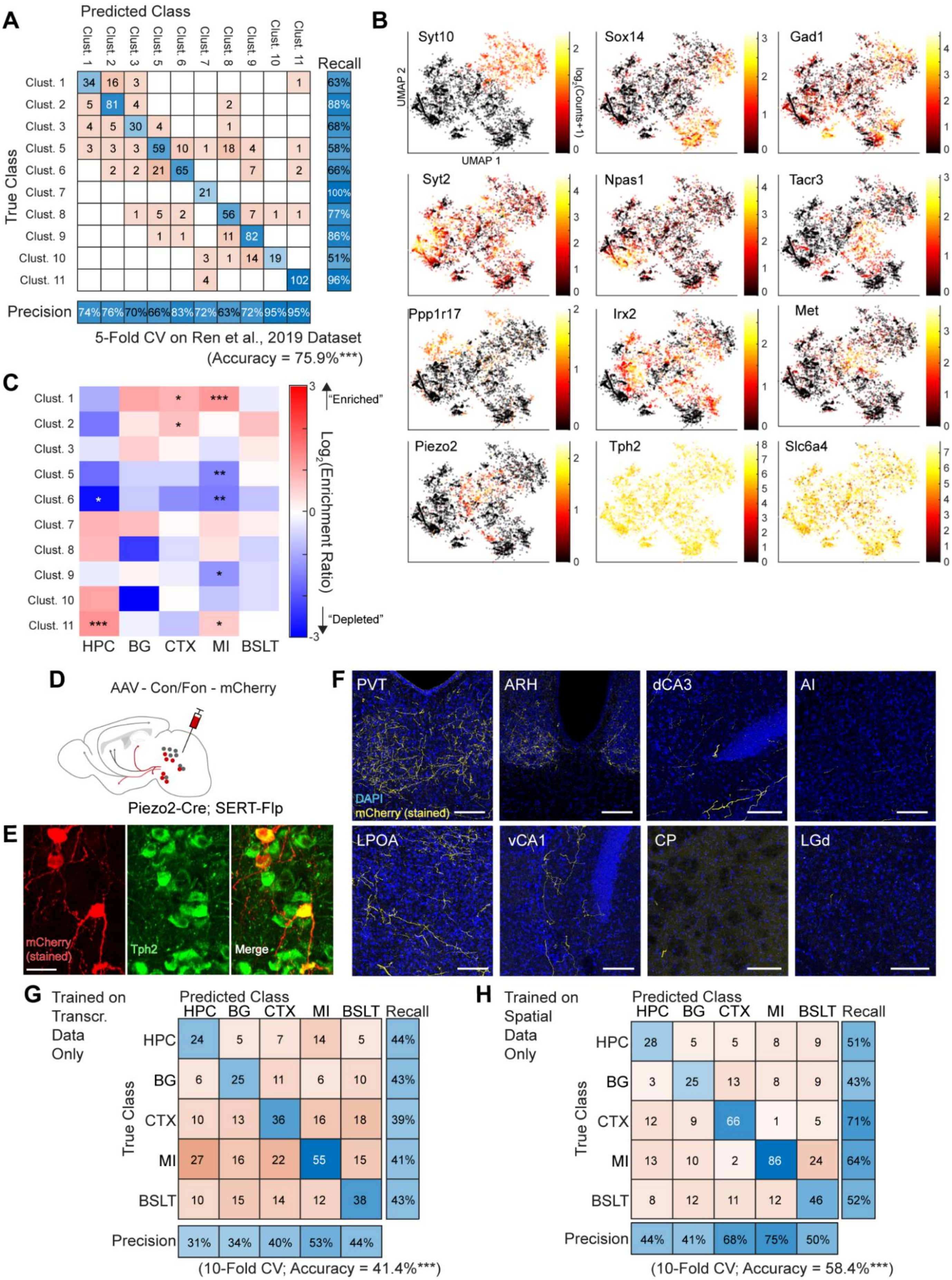

Figure S8. Spatial transcriptomic analysis using STARmap, related to Figure 5

bioRxiv preprint doi: https://doi.org/10.64898/2026.07.15.738594; this version posted July 17, 2026. The copyright holder for this preprint (which was not certified by peer review) is the author/funder, who has granted bioRxiv a license to display the preprint in perpetuity. It is made available under a CC-BY 4.0 International license.

(A) Validation of the consensus classification strategy on the published scRNA-seq dataset[25]. A classifier trained on the reference data was evaluated using 5-fold cross-validation, achieving 75.9% overall accuracy across 10 transcriptomic subtypes. Confusion matrix shows predicted versus true cluster assignments. ***p < 0.001, permutation test, 1,000 iterations (chance level = 12.3%).

(B) Expression levels of the 10 subtype marker genes across the 10 transcriptomic clusters identified by STARmap, confirming consistency with expected expression patterns from the reference dataset.

(C) Enrichment ratio (ER) heatmap showing the association between transcriptomic clusters and projectomic groups. Color indicates ER, defined as the proportion of a transcriptomic cluster among a group's barcode-positive cells divided by its proportion in the overall population. Red indicates enrichment (ER > 1); blue indicates depletion (ER < 1). Asterisks denote statistical significance (Fisher's exact test, FDR-corrected; *p < 0.05, **p < 0.01, ***p < 0.001).

(D) Intersectional validation strategy. A Cre/Flp-dependent mCherry reporter (AAV-Con/Fon-mCherry) was injected into the DR and MR of *Piezo2-Cre;SERT-Flp* mice to selectively label Piezo2-expressing serotonin neurons.

(E) mCherry-labeled neurons in the raphe co-express *Tph2*, confirming their serotonergic identity. Of 102 mCherry$^+$ neurons identified across 3 mice (2 females, 1 male), 90 (88.2%) were *Tph2*-positive. Scale bar, 25 μm.

(F) Projection targets of *Piezo2*$^+$ serotonin neurons. mCherry-positive axons were observed in the MI- (e.g., PVT, ARH, LPO) and hippocampal regions (e.g., dCA3, vCA1) but nearly absent in the projection targets of CTX, BG, and BSLT groups (e.g. AI, CP, LGd, respectively) in all 3 mice examined, consistent with the enrichment of Cluster 11 (*Met*/*Piezo2*-high) neurons in the HPC- and MI-projecting groups. Scale bars, 100 μm.

(**G**, **H**) Confusion matrices from 10-fold cross-validated random forest classifiers trained on transcriptomic features only (10 marker genes, *Tph2, Sert*; panel G) or spatial features only (AP, ML, DV positions, and DR/MR region; panel H). Class weighting was applied for unequal group sizes. ***p < 0.001, permutation test, 1,000 iterations (chance level = 20.0% for transcriptomic, 21.1% for spatial). Note that the cell-level spatial accuracy (58.4%) is lower than the animal-level distribution-based prediction (83.6%; **Figure 4C**), reflecting that single-cell spatial coordinates carry less information about projectomic identity than the full cell body distribution within an animal.

bioRxiv preprint doi: https://doi.org/10.64898/2026.07.15.738594; this version posted July 17, 2026. The copyright holder for this preprint (which was not certified by peer review) is the author/funder, who has granted bioRxiv a license to display the preprint in perpetuity. It is made available under a CC-BY 4.0 International license.

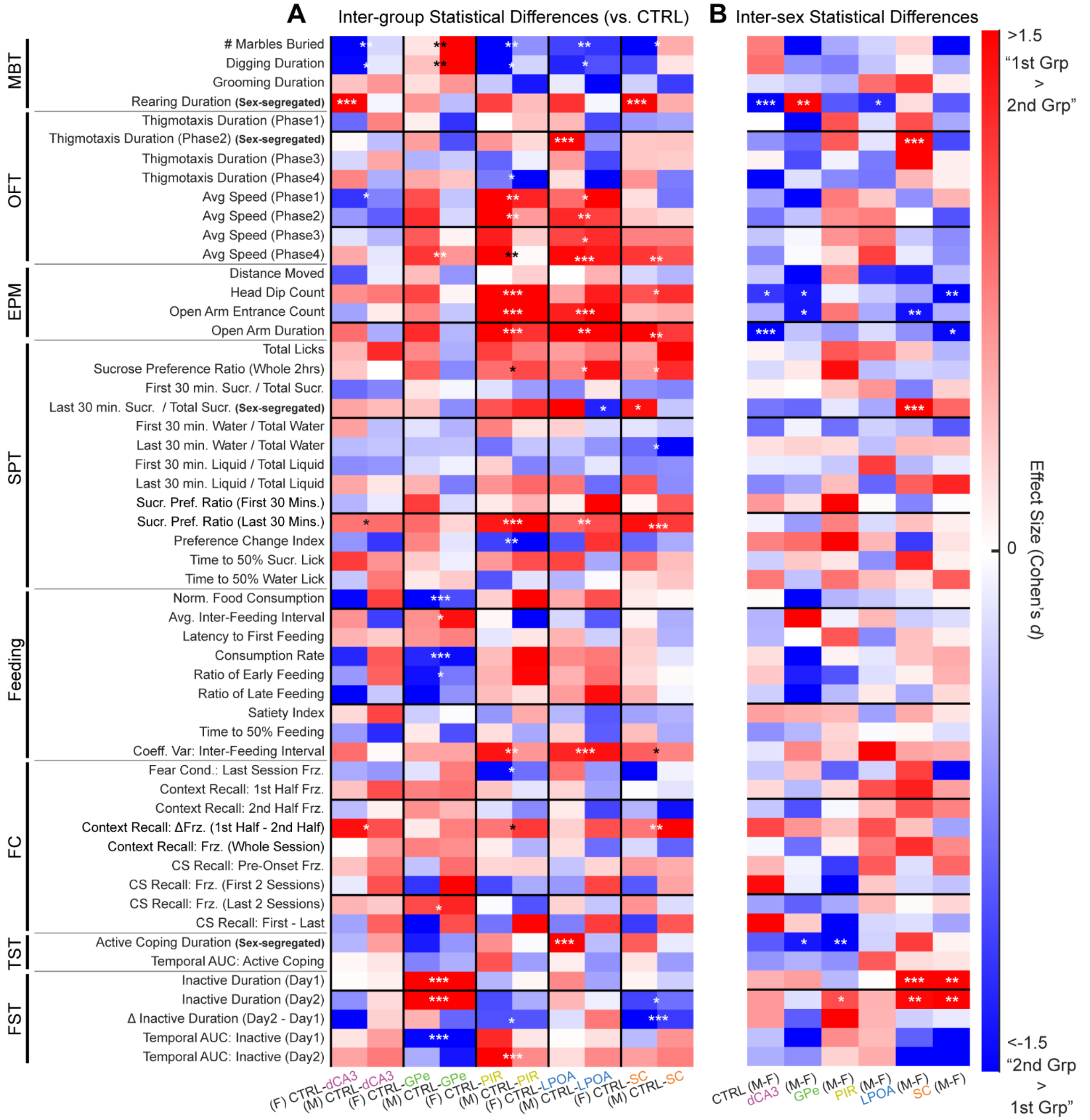

Figure S9. Statistical comparisons and effect sizes across 54 behavioral features, related to Figure 6

(A) Pairwise post-hoc tests against the control group were performed conditionally based on ANOVA results. See **Methods** for detailed descriptions of the 54 behavioral features. Inter-group comparisons were conducted only if a significant main effect of group was present ($p_{group} < 0.05$). If the interaction (sex × group) effect was significant ($p_{interaction} < 0.05$), analyses were stratified by sex (comparing experimental males with control males, and females with control females). If the interaction was not significant, sexes were pooled within groups. Regardless of statistical significance, Cohen's *d* effect sizes ($d = \frac{\mu_{1st\,group} - \mu_{2nd\,group}}{\sigma_{pooled}}$) are represented by a red-white-blue color scale to show the magnitude and direction of differences in a sex-specific manner. For example, the blue shading of "# Marbles Buried" in the "(F) CTRL-dCA3" column denotes that the female dCA3-injected

bioRxiv preprint doi: https://doi.org/10.64898/2026.07.15.738594; this version posted July 17, 2026. The copyright holder for this preprint (which was not certified by peer review) is the author/funder, who has granted bioRxiv a license to display the preprint in perpetuity. It is made available under a CC-BY 4.0 International license.

group buried more marbles than the female control group. BKY FDR-corrected across the 5 group-vs-control comparisons within each behavioral feature. *$p_{corrected}$<0.05; **$p_{corrected}$<0.01; ***$p_{corrected}$<0.001.

(B) Likewise, within-group sex differences were statistically tested only if the main effect of sex or the interaction effect was significant (p_{sex} or $p_{interaction} < 0.05$).

bioRxiv preprint doi: https://doi.org/10.64898/2026.07.15.738594; this version posted July 17, 2026. The copyright holder for this preprint (which was not certified by peer review) is the author/funder, who has granted bioRxiv a license to display the preprint in perpetuity. It is made available under a CC-BY 4.0 International license.

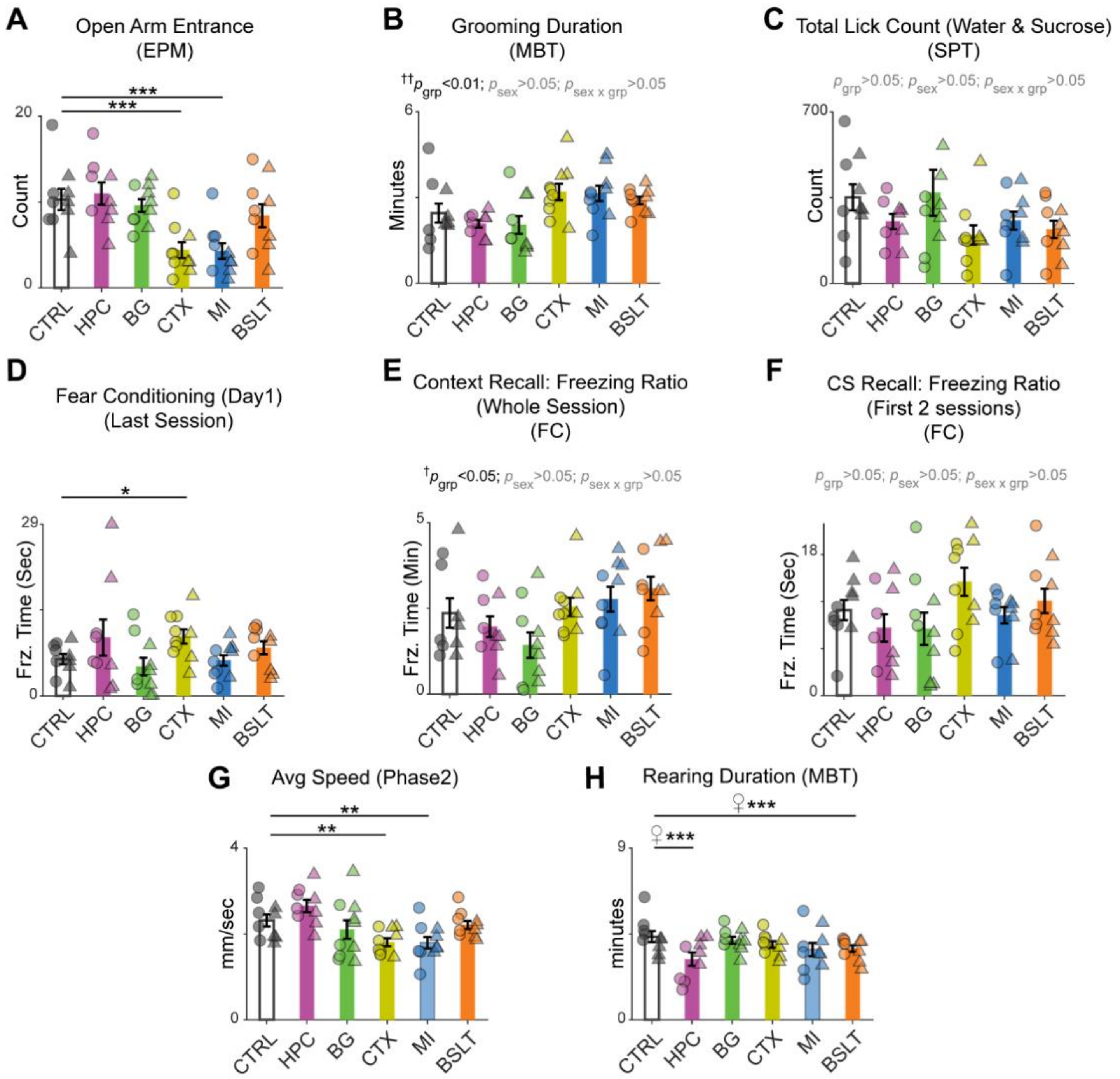

Figure S10. Extended behavioral characterization and feature correlation structure, related to Figure 6. **(A–H)** Additional representative behavioral features. Note that in Panel B, despite a significant group effect detected by two-way ANOVA, no pairwise differences relative to control were identified in the post hoc comparisons. In Panel H, sex-stratified pairwise comparisons were conducted as post hoc tests, as the interaction p-value was significant ($p_{\text{sex-group}} < 0.01$). *$p_{\text{corrected}} < 0.05$; **$p_{\text{corrected}} < 0.01$; ***$p_{\text{corrected}} < 0.001$; $^{\dagger}p_{\text{group}} < 0.05$; $^{\dagger\dagger}p_{\text{group}} < 0.01$

bioRxiv preprint doi: https://doi.org/10.64898/2026.07.15.738594; this version posted July 17, 2026. The copyright holder for this preprint (which was not certified by peer review) is the author/funder, who has granted bioRxiv a license to display the preprint in perpetuity. It is made available under a CC-BY 4.0 International license.

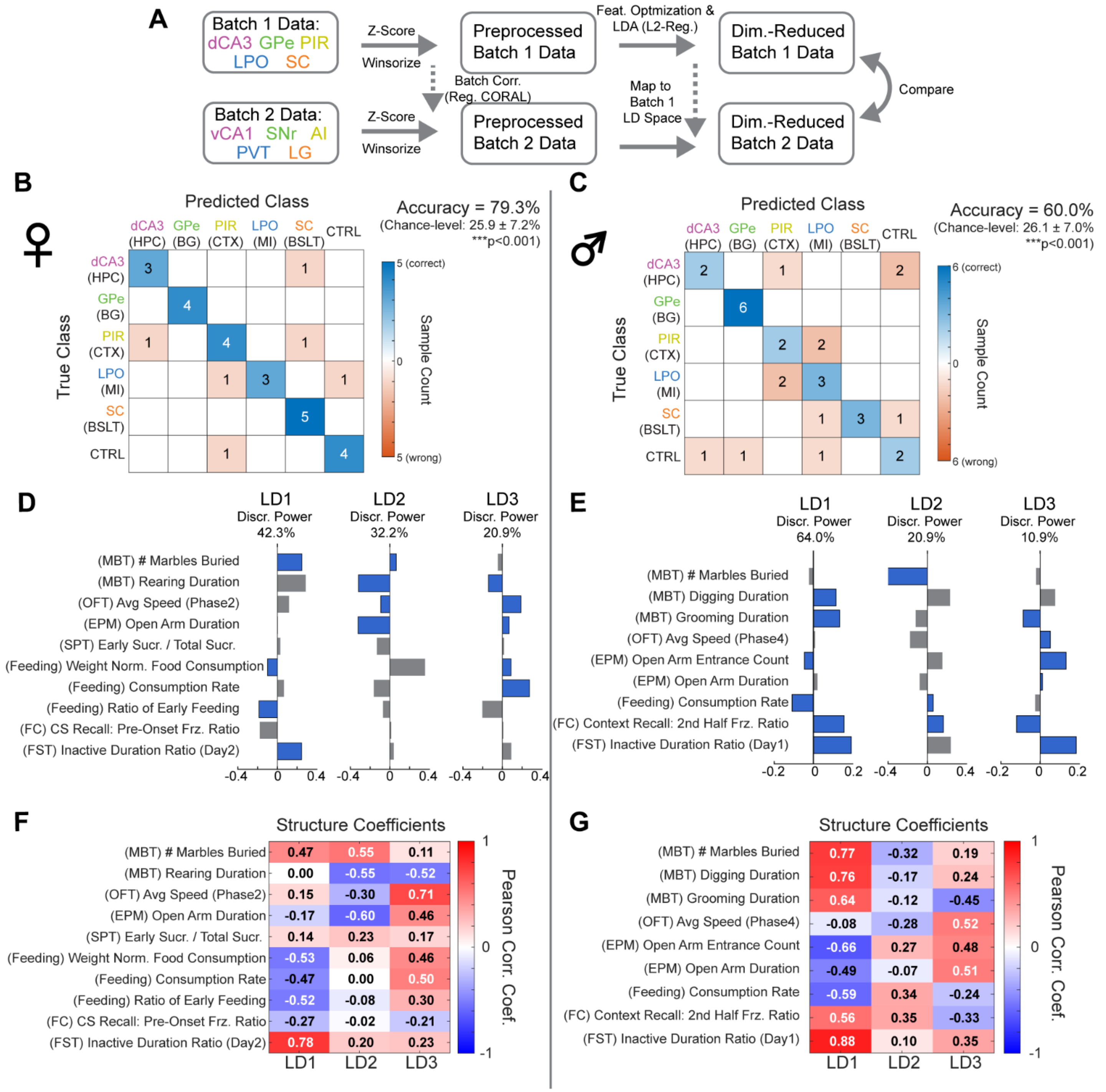

Figure S11. Supervised dimensionality reduction pipeline, model performance, and LD axis interpretation, related to Figure 7.

(A) Multidimensional model-based analysis pipeline. Dimensionality reduction was performed on Batch 1 data using L2-regularized LDA (top row). Using parameters derived from the Batch 1 model, Batch 2 samples were projected onto the Batch 1 LD space. See **Methods** for details.

(B, C) Performance of the model used for downstream analyses. A nearest-centroid classifier was applied to Batch 1 LD scores obtained using the selected feature sets and LD components. Validation was performed using leave-one-out cross-validation (LOOCV).

(D, E) A hyperparameter search to select optimal feature sets and the number of LD components identified three LDs for both sexes. Displayed are the features selected to effectively segregate groups, along with feature loadings derived from all Batch 1 samples using the optimized hyperparameters. Blue bars indicate features with

bioRxiv preprint doi: https://doi.org/10.64898/2026.07.15.738594; this version posted July 17, 2026. The copyright holder for this preprint (which was not certified by peer review) is the author/funder, who has granted bioRxiv a license to display the preprint in perpetuity. It is made available under a CC-BY 4.0 International license.

an absolute structure coefficient (shown in Panels F and G) greater than 0.3, suggesting a substantive association with the discriminant axis.

(**F, G**) Structure coefficients calculated as Pearson correlations between individual behavioral features and the LD scores. These coefficients quantify the direct association between each behavioral feature and LDs—independent of inter-feature correlations—and identify the primary behavioral drivers of group separation together with the LD loadings shown in Panels D and E.

bioRxiv preprint doi: https://doi.org/10.64898/2026.07.15.738594; this version posted July 17, 2026. The copyright holder for this preprint (which was not certified by peer review) is the author/funder, who has granted bioRxiv a license to display the preprint in perpetuity. It is made available under a CC-BY 4.0 International license.

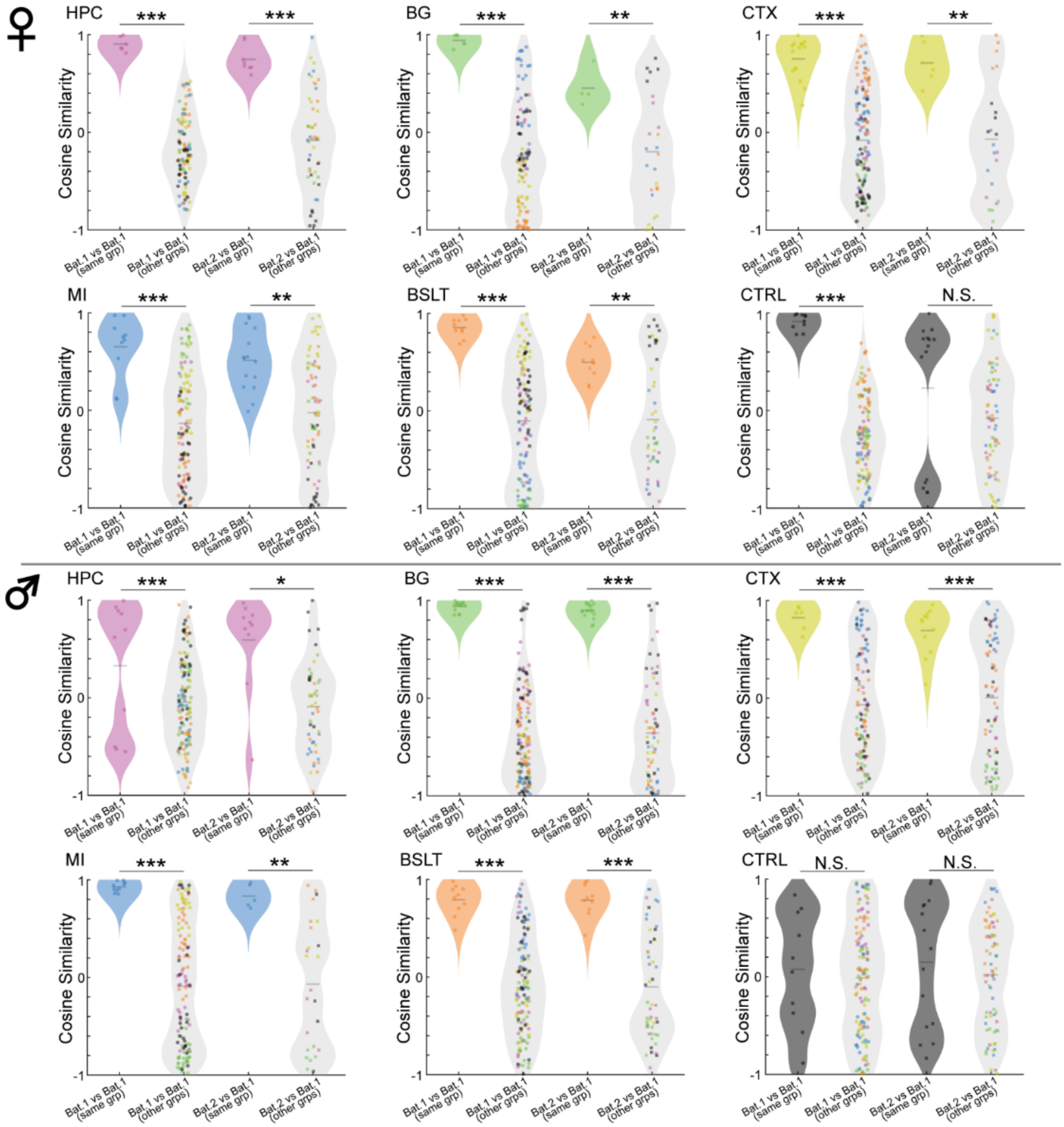

Figure S12. Within- and between-group cosine similarity of behavioral phenotypes across batches, related to Figure 7

Cosine similarity was computed from the linear discriminant (LD) scores shown in **Figure 7G** and **H**, to quantify how consistently serotoninergic projection-based groups cluster by behavioral phenotype within and across batches. Within-group similarity (colored violins) reflects pairs drawn from the same projection group, either within the same batch or across batches; between-group similarity (gray violins) reflects pairs drawn from the group of interest and samples outside that group. Higher within-group relative to between-group similarity indicates that serotonergic projectomic groupings generalize across batches. Each dot represents a single

bioRxiv preprint doi: https://doi.org/10.64898/2026.07.15.738594; this version posted July 17, 2026. The copyright holder for this preprint (which was not certified by peer review) is the author/funder, who has granted bioRxiv a license to display the preprint in perpetuity. It is made available under a CC-BY 4.0 International license.

pairwise cosine similarity value and is color-coded according to the projectomic group identity of the comparison sample (e.g., green dots indicate pairs involving BG-group samples). Within- vs. between-group cosine similarities were compared using a two-sided permutation test on the difference of means (n = 10,000 permutations). Multiple comparisons were corrected using the Benjamini-Hochberg FDR procedure. *$p < 0.05$, **$p < 0.01$, ***$p < 0.001$.

bioRxiv preprint doi: https://doi.org/10.64898/2026.07.15.738594; this version posted July 17, 2026. The copyright holder for this preprint (which was not certified by peer review) is the author/funder, who has granted bioRxiv a license to display the preprint in perpetuity. It is made available under a CC-BY 4.0 International license.

Movie S1. Fly-through videos showing whole-brain axon innervation patterns of two sample brains, related to Figure 1

Videos showing serial coronal sections from anterior to posterior for the two brains shown in Fig. 1C. Injection sites at dorsal striatum (left) and dorsal hippocampus (right) are indicated by *. CCFv3 (10-μm resolution) was upsampled to a 5-μm resolution and cropped to approximately match our imaging window. Then, 1,890 coronal virtual sections, each 5 μm thick, were overlaid with the processed axon volumes for visualization.

Table S1. Injection site coordinates and injection volume for axon tracing and behavior experiments, related to Figures 1, 6, and 7

Injection site coordinates and injection volume used to initiate axon tracing are provided. We note that the five injection sites in the CP were selected without prior knowledge of the results described in Fig. 3 but were chosen to ensure they were evenly distributed throughout the CP.

Table S2. Raw data for axon density and volume for 110 sample brains across 280 brain regions, related to Figures 1 and 2

Raw data and L2-normalized axon density and quantity are shown in separate sheets. Brain region abbreviations are given in Column B. Brain IDs are given in parentheses in Row 1.

Table S3. Probe sequences for STARmap, related to Figure 5

Probe sequences for marker genes and barcodes (Tab 1) and orthogonal reading (OR) probes (Tab 2) for spatial transcriptomic experiments using STARmap are provided. The sequences for *Gad1* were adapted from a previous study[118].

Table S4. Detailed information about retrograde virus encoding circuit barcodes, related to Figure 5

Information regarding retrograde viruses encoding circular barcode sequences, injection region combinations, and injection volumes is provided, with injection coordinates detailed in Table S1.

Table S5. STARmap expression, related to Figure 5

Retrograde barcode and marker gene puncta counts, individual cell coordinates, and the mouse and slice IDs of the cells used in the STARmap analyses are provided.

Table S6. Behavior quantification, related to Figures 6 and 7

Quantified behavioral features for all mice across the eight behavioral paradigms are provided. Mouse IDs, projectomic group assignments, sex, batch, and control/experimental designations are included.
